# Archival and Newly Isolated Historical *Bacillus anthracis* Strains Populate the Deeper Phylogeny of the A.Br.075(Sterne) Clade

**DOI:** 10.3390/pathogens14010083

**Published:** 2025-01-16

**Authors:** Markus Antwerpen, Peter Braun, Wolfgang Beyer, Dirk Aldenkortt, Michael Seidel, Gregor Grass

**Affiliations:** 1 Bundeswehr Institute of Microbiology (IMB), 80937 Munich, Germany; markusantwerpen@bundeswehr.org (M.A.); peter.braun@itmp.fraunhofer.de (P.B.); michael1seidel@bundeswehr.org (M.S.); 2 Institute of Infectious Diseases and Tropical Medicine, University Hospital Ludwig-Maximilian University Munich, 80799 Munich, Germany; 3 Fraunhofer Institute for Translational Medicine and Pharmacology (ITMP), Immunology, Infection and Pandemic Research, 80799 Munich, Germany; 4 Department of Livestock Infectiology and Environmental Hygiene, Institute of Animal Science, University of Hohenheim, 70599 Stuttgart, Germany; w-beyer@gmx.net; 5 Enviro Services International Sarl, Livange, 3378 Luxembourg, Luxembourg; aldenkortt@enviro.lu

**Keywords:** *Bacillus anthracis*, anthrax, phylogenetics, PCR assay, tannery, soil

## Abstract

The anthrax pathogen *Bacillus anthracis* can remain dormant as spores in soil for many years. This applies to both natural foci and to sites of anthropogenic activity such as tanneries, abattoirs, or wool factories. The A.Br.075 (A-branch) clade (also known as A.Br.Sterne) is prominent not only because it comprises several outbreak strains but even more so because spore preparations of its namesake, the Sterne strain, are counted among the most utilized anthrax animal vaccines. In this study, we genome-sequenced and analyzed 56 additional *B. anthracis* isolates of the A.Br.075 clade. Four of these we recently retrieved from soil samples taken from a decades-long abandoned tannery. The other 52 strains originated from our archival collection from the 20th century. Notably, the extended phylogeny of the A.Br.075 clade indicated that many of the newly added chromosomes represent basal members, some of which are among the most basal strains from this lineage. Twelve new strains populate a very deep-branching lineage we have named A.Br.Ortho-Sterne (also known as A.Br.076). A further 11 isolates amend the clade named A.Br.Para-Sterne (A.Br.078). Finally, some of the terminal clusters of the clade named A.Br.Eu-Sterne appear to be replete with (near) identical isolates, possibly a result of widespread use of the Sterne vaccine and of its re-isolation from vaccination-related animal anthrax outbreaks. From the accrued new phylogenetic information, we designed and tested a variety of new SNP-PCR assays for rapid and facile genotyping of unassigned *B. anthracis* genomes. Lastly, the successful isolation of live *B. anthracis* from a long-abandoned tannery reemphasizes the need for continued risk awareness of such sites.

## 1. Introduction

Endospores of *Bacillus anthracis*, the etiological agent of the zoonotic disease anthrax, can remain dormant in soil for years, possibly even decades [1,2,3,4]. Perturbing contaminated sites can result in resurfacing of infection-competent spores. Indeed, such instances have been reported [1,3]. Thus, soil acts as a longer-term reservoir for the anthrax pathogen both in natural environments including permafrost areas of high latitudes [4,5,6,7] or when industrial activities introduce *B. anthracis* into soil, e.g., at tanneries [8], abattoirs, or wool-processing facilities [9,10]. Such anthrax-foci, both natural or anthropogenic, represent sites of concern for environmental risk assessment as well as for human and animal health. At the same time, anthrax-foci offer research opportunities in countries in which anthrax is all but extinct. When *B. anthracis* can be retrieved from historical premises, this will make it possible to gain information on the former phylogenetic diversity of *B. anthracis* in areas where corresponding data had not been available before. Such explorative studies have been successful in the recent past. For instance, in Sweden [1], Belgium [10], and Germany [8], genetic information from the then-cultivated *B. anthracis* have been collected.

The small European country of Luxembourg is considered anthrax free [11,12]. Thus, there is no genomic information on the *B. anthracis* diversity in this central European region. For neighboring countries of Luxembourg, we have considerable information on prevalent genotypes only from France [13,14], but relatively little information from Germany [15,16], Belgium [10], or the Netherlands [17]. In spite of the lack of *B. anthracis* collections or outbreaks in Luxembourg, there are possibly unaccounted-for anthrax-foci in the form of remains from leather- or wool-processing industries in this country as well.

Spore preparations of the Sterne [18] strain of *B. anthracis* are in use as a live animal vaccine in many countries world-wide. This strain lacks one of the two virulence plasmids, pXO2 (encoding the capsule virulence factor), and thus only plasmid pXO1 (encoding the anthrax toxins) remains. Though considered an efficient vaccine, attenuated Sterne spores may rarely cause fatal infection after vaccination of livestock, e.g., of goats in southern African countries ([19] and personal communications with farmers in Namibia, 2017). From such diseased animals, Sterne will be re-isolated [20] and may be regarded as authentic infection. Moreover, the Sterne strain is also the namesake of one of the canonical phylogenetic lineages of *B. anthracis* [21,22]. This clade is known as A-branch (A.Br.) 001/002 (which is, as we know today, rather sensu lato Sterne) [22], A.Br.Sterne [21], or more systematically A.Br.075 (sensu stricto Sterne) [21]. Besides the Sterne strain, various other, including fully virulent A.Br.075 isolates, have been characterized [13,17,23,24,25]. Yet, databases are replete with variations of the Sterne vaccine genome [26], possibly as a result of re-isolation from vaccination failure.

In this study, we phylogenetically characterized 56 new *B. anthracis* strains via analysis of chromosome-wide single-nucleotide polymorphisms (SNPs). Four new isolates were retrieved from soil core samples from a derelict tannery in Luxembourg. The remaining 52 strains had hitherto been stored in our collection. Having retrieved new *B. anthracis* isolates, our aim was thus to amend and describe information on the genomic variability of this pathogen, rather than to provide epidemiologic information on *B. anthracis* in different ecosystems. All the genomes sequenced in this work could be placed within subclade A.Br.Sterne of the global *B. anthracis* phylogeny. From these genetic relationships, interconnections regarding the strains’ probable ancestry are discussed.

## 2. Materials and Methods

### 2.1. Collection of Soil Samples

In 2021, soil samples were collected from the site of the former waterworks area of an abandoned tannery near Luxembourg city during a test drilling down to a depth of about four meters below the soil surface. Layers of tanning pits and tanning barrels were identified and sampled, and horizons were stored at ambient temperature for further analysis.

### 2.2. Bacterial Culture, Inactivation, and Genomic DNA Preparation

*B. anthracis* isolates from our archival strain collection (Supplementary Table S1) were grown on Columbia blood agar (Becton Dickinson, Heidelberg, Germany) or trimethoprim–sulfamethoxazole–polymyxin blood agar (TSPBA) [3,27] at the Bundeswehr Institute of Microbiology biosafety level 3 (BSL-3) facility. *B. anthracis* cultures were chemically inactivated with 4% (*v*/*v*) Terralin PAA (Schülke and Mayr GmbH, Norderstedt, Germany), as in [28]. Genomic DNA was isolated using a MasterPure™ Gram Positive DNA Purification kit (Lucigen, Middleton, WI, USA) as described for Gram-positive bacteria. DNA concentrations were quantified using the Qubit dsDNA HS Assay Kit (Thermo Fisher Scientific, Dreieich, Germany) according to the supplier’s protocol. DNA preparations were stored at −20 °C until further use.

### 2.3. Soil Sample Analysis by PCR and Culturing of B. anthracis

Soil samples were processed for PCR analysis as described in [19] in the revised version published in [2]. In short, soil sample aliquots were mixed with sterile water and glass beads (Ø 5 mm) and were stirred overnight at room temperature. After rough filtration (through sterile gauze), centrifugation, and several wash-steps, the sample material was heated (65–70 °C) for 30 min to inactivate vegetative soil microbiota. After plating on semi-selective agar plates (TSPBA) [3,27] and incubation, the growth was collected into 0.9% (*w/v*) NaCl-solution. An aliquot of this suspension was boiled for 20 min in order to release DNA from cells and centrifuged, and then the supernatant was filtered through a 0.45 µm Luer-lock syringe filter. Aliquots of 5 µL of the filtrate were used for PCR analysis performed as real-time qPCR on *B. anthracis*-specific chromosomal markers *plcR* [3] or *dhp61* (*BA*_*5345*) [29] and, if required, plasmid markers *pagA* (pXO1) and *capC* (pXO2) [3]. If PCR-positive, dilutions of the original suspension were plated and grown on TSPBA [3,27] and checked for suspicious *B. anthracis* colonies.

### 2.4. Enrichment of B. anthracis from PCR-Positive Soil Samples by Magnetic Separation and Culturing

For enriching *B. anthracis* from soil samples that were tested positive for the anthrax pathogen by PCR, but failed to yield *B. anthracis* colonies, bead-assisted magnetic separation was applied as previously reported [2]. In brief, phage receptor binding proteins RBP_λ03Δ1-120_ or RBP_Wip1_ [28] were used to capture *B. anthracis* from enrichment cultures from positive soil samples (described in Section 2.2). First, Strep-Tactin XT protein (IBA GmbH, Göttingen, Germany) was coupled to magnetic beads (Dynabeads™ M-280 Tosyl-activated, ThermoFisher, Dreieich, Germany). Then, RBP_λ03Δ1-120_ or RBP_Wip1_ proteins were attached, and the RBP-loaded magnetic beads were utilized to separate *B. anthracis* from the cell-growth-containing liquid (from Section 2.2.) using a magnetic stand (ThermoFisher, Dreieich, Germany). Beads were washed with phosphate-buffered saline (PBS; pH~7.4) and finally plated onto TSPBA agar or Columbia blood agar plates (Becton Dickinson, Heidelberg, Germany). Colonies were PCR-tested for chromosomal *B. anthracis* marker *dhp61* [29], and positive isolates were stored at −80 °C.

### 2.5. Interrogation of SNPs via PCR by Relative Ct-Value Analysis (Delayed Mismatch Amplification PCR Assay, DMAA)

De novo designed or adapted from previous work [30], Delayed Mismatch Amplification PCR Assays (DMAA) [15,31] were employed to experimentally interrogate for clade-specific SNPs. These SNPs were identified by whole-genome SNP analysis and used to determine the distribution of these SNPs in additional *B. anthracis* DNAs. This probe-free, real-time qPCR assay interrogates the character state of SNPs (derived, DER vs. ancestral, ANC) in two separate PCR reactions containing matching and mismatching forward primer oligonucleotides preferentially favoring amplification of one of the two alleles and a common reverse primer oligonucleotide. PCR runs are scored by simple relative subtraction of numerical PCR threshold values, i.e., by subtracting the numerical Ct value of the ANC allele from that of the DER allele (Δ(Ct_DER_–Ct_ANC_). The forward primer matching the template DNA at its 3’-end will amplify more efficiently (i.e., yield a lower Ct value) than the one featuring the other allele, and this reaction will thus determine the SNP group [15]. DMAA SNP-primer oligonucleotides for new SNPs and established canSNPs were designed surrounding the SNP positions using the Geneious Prime software (Dotmatics, Bishops Stortford, United Kingdom) with the *B. anthracis* Ames ‘Ancestor’ chromosome (accession number NC_007530) as a (ancestral) reference. DMAA-SNP primer sequences for real-time PCR assays are listed in Supplementary Table S2. Each primer pair was used in a 20 μL single-plex reaction. For this, 1 μM of each primer pair and approximately 20 ng of the template DNA were added to 1× LightCycler 480 High Resolution Melting Master mix (Roche, Mannheim, Germany). Amplification and Ct-value analysis without melting curve analysis was carried out on a LightCycler 480 II instrument (Roche, Mannheim, Germany) as described in [32].

### 2.6. Whole Genome Sequencing

Genomic libraries of *B. anthracis* DNAs were constructed using NEBNext^®^ Ultra™ II DNA Library Prep Kit (New England Biolabs, Frankfurt am Main, Germany) or Illumina DNA Prep (Illumina, Berlin, Germany) with 100 ng of input DNA. Subsequent use of the Illumina MiSeq platform with 2 × 300 bp v3-chemistry produced at least 300,000 reads for each isolate. Alternatively, the NextSeq 2000 system (New England Biolabs, Frankfurt am Main, Germany) was used with 2 × 150 bp P2 chemistry producing at least 300,000 reads for each sample.

For four strains, DNA was sequenced using IonXpress Plus fragment library preparation with size selection in combination with the IonTorrent PGM instrument (Life Technologies, Carlsbad, CA, USA) according to the manufacturer’s recommendations. The resulting raw reads had the characteristics of 300 bp single-end reads.

All sequence reads were assembled de novo using an in-house script based on the SPAdes (version 3.15.3) assembler [33] to create draft genomes. Pilon (version 1.24) [34] was used to correct SNPs or to close small gaps and INDELs. The *B. anthracis* canSNP-group was determined for these polished scaffolds using canSNPer-Software (V5)(10.1093/bioinformatics/btu113, accessed on 30 January 2024) in combination with canSNPer2-Database (https://github.com/FOI-Bioinformatics/CanSNPer2-data, accessed on 30 January 2024). All data generated or analyzed during this study are included in this published article, and their supplementary information is publicly available in the NCBI Sequence Read Archive (SRA) repository; Bioproject PRJNA309927.

### 2.7. Analysis of Whole Genome Sequencing Data and SNP-Calling

Representative genomes and scaffolds of *B. anthracis* belonging to the A.Br.075 canSNP group were downloaded from the ncbi SRA-database or genome repository and assembled (as described above in Section 2.4) if needed. All of these chromosomes were aligned (parameters -c -e -u -C 1000) using the Parsnp tool of the Harvest Suite (version 1.1.2) [35], against the reference chromosome of *B. anthracis* ‘Ames Ancestor’ (NC_007530), which also served as the phylogenetic outgroup. HarvestTools (version 1.2) of the same software suite [35] was used to export these SNP positions and to create a variant calling file (vcf) for further analysis of the identified SNP positions. In order to avoid biased estimations and enhance data quality, chromosome regions with closely adjacent SNPs (<10 bp distance) and positions harboring undefined nucleotides (‘N’) were masked prior to further analyses. Using this curated vcf-file (Supplementary Table S3), a multi-FASTA-file was compiled from the chromosome dataset comprising the concatenated 957 SNPs as a multiple-sequence alignment. This concatenated sequence information was the final framework to calculate a set of most-parsimonious trees using the parsimony ratchet [36] with nearest neighbor interchange rearrangement via the software R (4.3.3.) in combination with the package Phangorn (V2.12.1) [37]. The resulting Newick-tree was exported and loaded into the Grapetree tool (1.5.0.) [38] for visualization and subsequently manually edited (using Powerpoint 2016, Microsoft) for optimization of style and display of associated metadata.

## 3. Results

### 3.1. Live B. anthracis Could Be Isolated from Soil Samples of an Abandoned Tannery Site in Luxembourg

**Figure 1:**
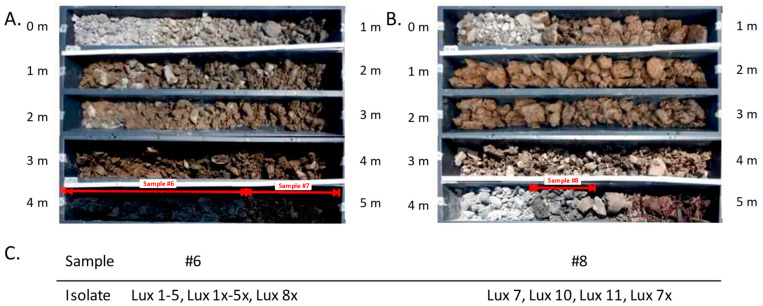
Partial horizons of drillings for sample acquisition at a derelict tannery and isolates thereof. Partial horizons of boreholes from the waterworks area and tanning pits or tanning barrels are shown. (**A**) Samples #6 and #7 from 4.0–4.7 or 4.7–5.0 m depth, respectively, composed of backfill, organic matter (low density), and decomposed wood (blackish). (**B**) Sample #8 from 4.3–4.5 m depth composed of fill, organic matter, decomposed wood, and animal skins (dark brown). Indicated by red arrows are core sample numbers yielding *B. anthracis*-positive *plcR* PCR results (#6 and #8) and a sample that turned out negative upon further analysis (#7). (**C**) Designations of isolates retrieved from core samples #6 and #8.

Samples of soil cores taken from a depth of 0 to 5 m were processed, and spores were cultivated on solid media. Partial horizons of the drillings related to these soil cores are shown in Figure 1A,B. Three out of ten samples from these horizons yielded mixed microbial growth and were PCR positive for the chromosomal *B. anthracis* marker *plcR*.

However, further repeated efforts to isolate *B. anthracis* directly from this mixed microbial culture by dilution plating and colony morphology analysis remained unsuccessful. Therefore, an alternative enrichment approach was chosen. For this, recombinant receptor-binding proteins (RBP) of *B. anthracis* (pro)phages coupled to magnet beads were employed, which bind specifically to *B. anthracis* cells after spores were allowed to germinate. A total of 15 candidate colonies (Figure 1C) were confirmed by PCR with chromosomal marker *dhp61* as *B. anthracis* and further analyzed. Each of these isolates also possessed both virulence plasmids (pXO1 and pXO2). Isolates with a number designation followed by ‘x’ were isolated using the RBP of phage Wip1, those with plain numbers using RBP BA4079 (Figure 1C).

### 3.2. Preliminary Genotyping by DMAA canSNP-PCR Groups the New Soil Isolates to the A.Br.001/002 canSNP Clade

**Figure 2:**
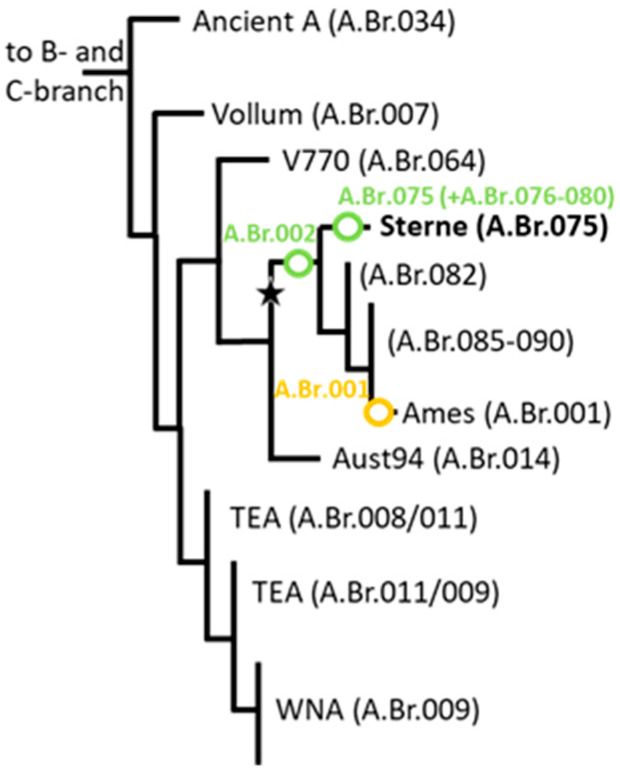
Schematic overview of the phylogeny of *B. anthracis*. Shown is a simplified phylogenetic tree focusing on the A-branch, drawn (not to scale) from data in [21]. Colored in black are major canSNP clades of *B. anthracis*. Light green circles denote the position of canSNPs defining lineages of the new genomes sequenced in this study. Indicated in orange is the canSNP that leads to the reference (Ames ‘Ancestor’). The star designates the ancestral lineage of both A.Br.001 and A.Br.002 clades (identical to that shown in Figure 3).

In order to determine the diversity of these new soil isolates, we applied DMAA canSNP genotyping using PCR assays that we adapted from the first-generation of *B. anthracis* canSNP PCR tests [22,30]. Figure 2 provides a schematic overview of the overall phylogeny of *B. anthracis* for orientation. PCR results yielded ancestral alleles for the most basal B-branch (B.Br.003) assay and for A.Br.001–009, except for A.Br.002 (Table 1; exemplary shown for canSNP A.Br.001 vs. A.Br.002). All 15 tannery isolates showed derived alleles for canSNP A.Br.002 (Table 1) and thus grouped to the A.Br.Sterne clade. Previously genotyped DNA of strains from our collection that either featured an ancestral allele for canSNP A.Br.001 or a derived allele for canSNP A.Br.001 served as architype derived or ancestral genotype samples, respectively. Their averaged DMAA-PCR values are summarized in Table 1.

### 3.3. All Newly Sequenced Genomes Group Within the A.Br.075(Sterne) canSNP Clade Phylogeny

Next, we isolated DNA from archival strains of our collection previously assigned to A.Br.002, more specifically to A.Br.075(Sterne), the latest, most closely defined canSNP for the Sterne clade [21]. We then sequenced the genomic DNA of the SNP-identified isolates (including 4 randomly selected isolates out of the 15 new ones from tannery soil described in Table 1) and mapped the chromosomal reads to the *B. anthracis* Ames ‘Ancestor’ reference chromosome. All our new genomes indeed grouped within the A.Br.075 clade. Further analysis of these and of additional chromosomal sequences from public databases belonging to the A.Br.075 clade produced a total of 957 informative chromosomal SNP positions (Supplementary Table S3). Figure 2 indicates the position of the A.Br.075(Sterne) clade within the global phylogeny of *B. anthracis*.

### 3.4. Three Distinct Lineages Within the A.Br.075(Sterne) canSNP Clade Include All Newly Sequenced Genomes

**Figure 3:**
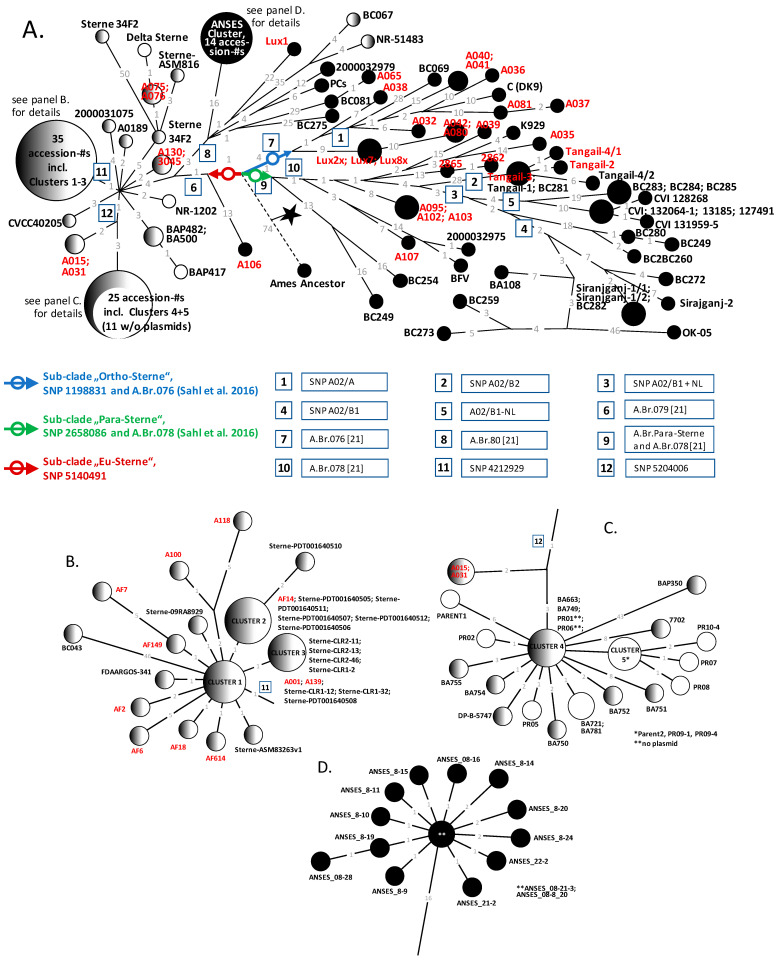
Maximum parsimony tree (MPT) of the canonical A.Br.075(Sterne) clade of *B. anthracis*. An MST was calculated from 957 chromosomal SNPs and visualized using Grapetree [38] (**A**). Numerical SNP distances between nodes (chromosomes) and branching points are indicated at the branches (as grey numbers). Newly sequenced isolate names are displayed in light red, and sub-clade-defining SNPs in dark red (A.Br.Eu-Sterne), blue (A.Br.Ortho-Sterne), or green (A.Br.Para-Sterne) arrows with circles. Clusters of closely related isolates compiled as large circles in panel A are shown in detail in panels (**B**–**D**). Designations of additional informative SNPs are highlighted as numbers in boxes (respective chromosome positions are listed in Supplementary Table S3). Filled, partially filled, and empty circles indicate the presence of both plasmids either pXO1 (filled left) or pXO2 (filled right) or no plasmid, respectively.

A minimum spanning tree (MST) tree calculated from the chromosomal SNP data (Supplementary Table S3) illustrates the position of every newly sequenced chromosome and all other accessible *B. anthracis* chromosomes featuring the A.Br.075 (Sterne) genotype from public databases (Figure 3). A prominent feature of this tree was three branches emanating from the root of this canSNP group (blue, red, and green arrows in Figure 3). This sub-branching node was 74 SNPs distant from the Ames ‘Ancestor’ reference. We named one sub-clade A.Br.Ortho-Sterne (with one out of four defining canSNPs at chromosome position 1,198,831; blue arrow and circle in Figure 3; also canSNP A.Br.076 [21] defines this branch). The second basal branching gave rise to sub-clades A.Br.Para-Sterne (with its single canSNP at chromosome position 2,658,086; green arrow and circle in Figure 3; also canSNP A.Br.078 [21] defines this branch and SNP). Lastly, the branch ‘Eu-Sterne’ features a single basal SNP at chromosome position SNP 5,140,491 (red arrow and circle in Figure 3). This SNP was one SNP proximal to canSNP A.Br.079 [21] (boxed-in number 6 in Figure 3).

### 3.5. Most Newly Sequenced Isolates Including Three from Tannery Soil Populate Sub-Clade A.Br.Ortho-Sterne

Sub-clade A.Br.Ortho-Sterne contained the least number of strains in any of the three clades in our analysis (Figure 3), but it is replete with newly sequenced strains. The maximum SNP distance from root to tip was 40 SNPs (strains 2000032879 and BC069), and the maximum between any two strains from this clade was 43 SNPs (strains 2000032879/BC069 and A037). Notably, most of the strains (12 out of 15) were those we newly sequenced in this study. Among these, three out of the four genome-sequenced tannery soil isolates also grouped within this lineage. All three were SNP identical, thus most likely representing a single clone. This likely clone was a mere 13 SNPs distant from the base of the A.Br.Ortho-Sterne sub-clade (and thus the branching point to the outgroup). Remarkably, these tannery soil isolates were ancestral to two strains from our historic collection (A042 and A080), separated by 10 SNPs (Figure 3). Strains A042 and A080 were isolated in Germany, possibly from dead cattle at an unknown time but likely before 1971.

### 3.6. Only One Sequenced Tannery Soil Isolate Group Within Sub-Clade A.Br.Eu-Sterne

The maximum SNP distance in sub-clade A.Br.Eu-Sterne from root to tip was 61 SNPs (strain Sterne 34F2, Accession-# ASM200526v1), and the maximum between any two strains from this clade was 103 SNPs (strains Sterne 34F2 and BAP350) (Figure 3). Sub-clade A.Br.Eu-Sterne comprises not only all Sterne sensu stricto strains derived from the Sterne live vaccine and a number of fully virulent wild isolates (black circles in Figure 3) but also a single tannery isolate (Lux1) (Figure 3). This isolate featured 22 autapomorphous (unique for this isolate) SNPs rooting Lux1 to a small polytomy and separating the isolate from sister lineages leading (i) to a group of nearly identical strains (ANSES Cluster; Figure 3D) featuring 16 synapomorphous, shared SNPs), (ii) to two strains of unknown origin (BC067 and NR-51483, with 35 synapomorphous, shared SNPs), (iii) to two strains (2000032979 and PCs, featuring four synapomorphous SNPs), and (iv) to a group of three strains (A065, BC081, and BC275, featuring 1 synapomorphous SNP Notably strains BC081 and A065 differed by one SNP only. BC081 was isolated in the USA from an animal bone, whereas A065 was likely isolated from dead cattle in Germany (possibly in the 1950s and 1960s) (Figure 3A). This clearly suggested a common origin of the imported *B. anthracis* contamination across continents.

Of note, a single newly sequenced strains from our archival collection, A106 from Germany isolated in 1962 (featuring 13 autapomorphous SNPs), branched off directly after the root of sub-clade A.Br.Eu-Sterne and before canSNP A.Br.079 [21]. Thus, this isolate constituted the hitherto most basal branching single isolate of the entire lineage (Figure 3).

### 3.7. The Majority of Strains from Sub-Clade A.Br.Eu-Sterne Are Lacking Virulence Plasmid pXO2

All other newly sequenced strains from our archival collection were distributed across many branches of a polytomy six SNPs distal from the Eu.Sterne canSNP or subsequent secondary polytomies (Figure 3). Each of the strains from our collection (from various origins) had been categorized as ‘Sterne’, all lacking plasmid pXO2. In fact, none of the strains positioned between canSNP A.Br.079 and A.Br.080 [21] carried plasmid pX02 (14 of them even lacked both virulence plasmids).

Notably, strains AF2, AF6, AF7, AF18, AF149, and AF614 also lacked plasmid pXO2. These strains had been collected from diseased caprines or water samples (AF149) in Namibia from 2009 to 2013. Thus, it is very likely that each of these (except for AF149) might have been collected as the result of *B. anthracis* isolation from dead animals subsequent to vaccine failures. The reason for such failure is incompliance with the rules for vaccine administration to goats as generally provided by the vaccine producers. Such rules include the reduction of the dosage for goats and changing vaccination sites from the neck to the tail root of the animal, whereby deaths caused by edemas at the throat region are prevented. Notably, these Namibian AF isolates were not SNP-identical. There were between two and eleven SNP differences among these strains, suggesting heterogeneity within the administered Sterne vaccines.

### 3.8. Most Newly Sequenced Archival Isolates of Sub-Clade A.Br.Para-Sterne Group Within Basal Lineages

A common feature of most newly sequenced archival strains located within the A.Br.Para-Sterne sub-clade was that these isolates branched off close to the name-sake canSNP (Figure 3). Overall, the maximum SNP distance in sub-clade A.Br.Para-Sterne from root to tip was 68 SNPs (strain OK-05), and the maximum between any two strains from this clade was 98 SNPs (strains BC249 or BC254 and OK-05).

Among the newly sequenced strains, the pair 2862 and 2865 or the triple A095, A102, and A103 were very likely clonal. Strains A035, A039, and A107 populated two previously existing minor branches off the main lineage, while the pair 2862 and 2865 as well as the triple A095, A102, and A103 formed their own minor branches (Figure 3). Notably, the most basal lineages of sub-clade A.Br.Para-Sterne exclusively featured strains that have most likely been isolated as a result of importation from a different world region. The geographic distribution of newly sequenced strains is discussed in more detail below.

### 3.9. Additional B. anthracis Strains of the A.Br.075 Clade Can Be Genotyped by New DMAA SNP-PCR Discriminatory Assays

In addition to the four genome-sequenced tannery isolates (Lux 1, Lux7, Lux2x, and Lux8x), we retrieved nine more *B. anthracis* isolates from the respective soil samples (Figure 1C). In order to place these nine isolates within the A.Br.075 phylogeny without further genome-sequencing efforts, we designed and tested new DMAA assays. Since already three of the genome-sequenced isolates were SNP identical, chances seemed high that more of these isolates were also SNP identical clones. Three new DMAA PCR assays were named after their namesake SNPs A.Br.Eu-Sterne, A.Br.Ortho-Sterne, and A.Br.Para-Sterne located at the root of their respective lineages (Figure 3; Supplementary Table S2). DNA of strains from our collection served as derived or ancestral genotype architype samples. These assays were then utilized to interrogate for the SNP states of the surplus soil isolates (from Figure 1C) and to validate the SNP states of strains from the institute’s collection including the four genome-sequenced tannery soil isolates (Table 2).

Notably, all additional tannery soil isolates grouped within the A.Br.Ortho-Sterne sub-clade and were thus likely clonal to the triple Lux7, Lux2x, and Lux8x (Table 2, Figure 3). This is not entirely surprising since all isolates were enriched from mixed growth on cultured agar plates derived from soil samples (Section 3.1). We also designed additional DMAA PCR assays for several sub-clades of the A.Br.075 phylogeny. These new assays interrogate for the previously identified canSNPs A.Br.079 and A.Br.080 [21], the informative SNP A02/B1 + NL [17], and SNP positions 4,212,929 as well as 5,204,006 (from this work). From subsequent DMAA-PCR analysis, the SNP states of several (genome-sequenced) strains from our collections (previously SNP interrogated by other means) were confirmed (Table 2). Thus, these easy-to-implement DMAA SNP PCR assays will provide other researchers without access to genome sequencing the opportunity to genotype their A.Br.075 isolates.

## 4. Discussion

Prior to the wide-spread use of genome sequencing, it was recognized that the A.Br.075(Sterne) lineage (by then known as clade A3.b, together with A.Br.Ames) was one of the most dominant genotype(s) of *B. anthracis.* We had been well informed about the most likely regions of the world where this lineage was autochthonous. These regions comprise many East Asian countries such as China [39] Vietnam [40], Indonesia [40], and Bangladesh [25]. In the south of the USA (Texas), a branch of A.Br.Ames has also become firmly established [41]. In Europe, the A.Br.001/002 clade has caused outbreaks in, e.g., the United Kingdom, the Netherlands, Germany, Belgium, Denmark, and France [17,42].

Unfortunately, many of the strains genotyped with older techniques have not yet been sequenced at the genome-level genome. Thus, the diversity of the A.Br.Sterne lineage is very likely much higher than we observe in the study at hand or elsewhere. Today, canSNP clade A.Br.002 splits up into canSNP sub-clades A.Br.075(Sterne) and A.Br.081(A.Br.Ames), as well as the remainder of canSNP clade A.Br.001/002 (which is now canSNP group A.Br.082) [21,43]. In China, the sister canSNP clade A.Br.082 of A.Br.075(Sterne) and A.Br.081 (A.Br.Ames) was found to be one of the most prevalent [39,43,44,45]. Several lineages outside the A.Br.075(Sterne) clade can be found in Russia and Kazhakstan [46]. In Russia, canSNP clades A.Br.081(A.Br.Ames) and A.Br.082 are common [43], while A.Br.075(Sterne) is not.

Of note, while our collection harbored a considerable number (>14 uniques) of fully virulent A.Br.075 isolates, there were no other A.Br.001/002 or A.Br.Ames strains in our collection (except the Ames namesake and the two isolates from two German bovine anthrax cases in 2012 and 2014). We find this (near) absence remarkable because both A.Br.Sterne and A.Br.Ames strains can be found in, e.g., China [39] and Siberia [46], or as imports in diverse European countries [13,17,23] or the USA [41].

Regarding our archival A.Br.Sterne isolates of canSNP group Ortho-Sterne (A.Br.076) (Figure 3A), strains A032, A036, A037, A038, A040, A041, and A081 were all most likely isolated from cattle in Germany between the late 1950s and early 1960s (Supplementary Table S1). Strains A040 and A041 were most likely clonal from the same origin. The only other two previously sequenced strains in this sub-clade (C [DK9] and 2000032879) were from Denmark or Pakistan, isolated in 1960 and 1962, respectively. All the strains in this canSNP group are likely imports into the respective countries in which they had been isolated. Similarly, the tannery isolates Lux2x, 7, and 8x as well as isolates A042 and A080 (likely isolated from a diseased cow in Germany between the late 1950s and early 1960s) are likely imports.

Also, from canSNP group Para-Sterne (A.Br.078) isolates 2862 and 2865 (isolated in Germany between 1960 and 1970), the identical isolates A095, A102, and A103 (isolated in Germany, in 1962, 1954, and 1962, respectively), and isolates A107 (from Germany, 1962), BFV (from Jamaica, unknown time of isolation), and 2000032975 (unknown country of origin, from 1966), can be considered imports. Notably, strains A035 (Germany, 1968) and K929 (Denmark, 1966) share a recent ancestor and thus a common region from which they were imported into Europe (Figure 3A).

Conversely, newly sequenced isolates Tangail-2, Tangail-3, and Tangail-4/1 from sub-branch A02/B2 can be considered autochthonous *B. anthracis* from Bangladesh. These three, together with the previous isolated strains Tangail-1 and Tangail-4/2 [25], form a closed clade separated by 28 synapomorphous SNPs from the rest of canSNP group Para-Sterne (A.Br.078).

Two of our now sequenced isolates were positioned within canSNP group A.Br.080. Lux1 is a new tannery soil isolate, and strain A065 has been isolated in Germany (possibly in the 1950s and 1960s). All the now-sequenced strains in canSNP groups A.Br.076, A.Br.078, and A.Br.080 are fully virulent (harboring broth virulence plasmids pXO1 and pXO2). Also, the majority of the remainder of strains in these canSNP groups harbor both plasmids as well (Figure 3A). This is in contrast to strains in large sections of canSNP group A.Br.079 (Eu-Sterne), which lack plasmid pXO2 (or even both plasmids). Remarkably, however, clade A.Br.080, which is a A.Br.079 sub-clade, features mostly fully virulent isolates (with both plasmids). This very basal sub-clade A.Br.080 branched off a mere two SNPs distal to the polytomy at the base of A.Br.075 (Figure 3A). In the canSNP group A.Br.079 (Eu-Sterne), we added new sequences of SNP-identical pairs A001/A139, A015/A31, A075/A076, and A130/3045. These had been designated as ‘Sterne vaccine-like strains’ in our archival strain collection lacking plasmid pXO2. Other singletons (A100 and A118) also featured plasmid pXO1 only.

Strains AF2, AF6, AF7, AF18, AF149, and AF614 were collected in Namibia from 2009 to 2013. All grouped within the A.Br.Eu-Sterne group. Every one of these yielded a unique SNP genotype, though most were either grouping within cluster 1 to 3 or where only 1 SNP distant from these. Exceptions were AF2, AF6, and AF7 that were two, five, and five SNPs apart (Figure 3B).

Finally, the most basal branching isolate from sub-clade A.Br.Eu-Sterne was strain A106 (Figure 3) from Germany isolated in 1962. This most probably imported strain features a complete set of plasmids and can therefore be considered fully virulent.

In addition to strains from recent outbreaks, archival strain collections [22] or inactive historical specimens of *B. anthracis* [16,21], and also historically anthrax-contaminated sites, may provide valuable phylogenetic information for this pathogen [8,10,47]. While valuable per se for information related to the historical distribution of genotypes at the investigated location, care has to be taken when correlating recovered historical genotypes with the previous autochthonous presence of *B. anthracis* in the geographic area of investigation [48]. This is largely because of anthropogenic activities that have aided in the almost worldwide spread of viable *B. anthracis* spores over many centuries. Correct predictions of anthrax phylogeography can thus be reconstructed best if there is at least some data of the previous extant local diversity of the anthrax pathogen. On the other hand, it can possibly be judged by exclusion whether a given genotype is most likely introduced from other world regions and is not locally autochthonous. In a study on the phylogeny of *B. anthracis* in France, the authors noted that the French A.Br.001/002 clade isolates appeared to be quite dissimilar and thus less related to one another. From this observation, the authors could therefore not exclude several distinct introductions into France and subsequent differentiation within this canSNP group [13].

A relatively well-documented, enduring anthrax soil focus resulting from tannery activities has been reported from the Netherlands [17]. It has resulted in dead animals reaching from 1997 possibly back to 1886. Since spores can remain in soil for extended times, it is unsurprising that they can also endure in (surface) waters and mud (including tannery wastewater), as well as on hides, fur, wool, hair, and other products from diseased or slaughtered animals [49]. As pointed out earlier, *B. anthracis* could be retrieved from dried soil after being stored for sixty years [50]. We have previously isolated DNA from a non-viable anthrax specimen from 1878 but no viable spores [16]. Very recently, viable spores were retrieved from the remains of a former WW II era bacteriology laboratory site in China after about 75 years of dormancy [51]. Even more remarkably, viable bacteria were cultivated from 200 year (± 50) old bones collected during archeological excavations in South Africa [52].

Indeed, while considered a neglected tropical disease, anthrax may also cause major outbreaks in (higher) northern latitudes. In particular, one recent outbreak in reindeer in Siberia (Yamal-Nenets tundra region) in 2016 is noteworthy. This outbreak killed approximately 2400 reindeer, led to the hospitalization of about 100 local residents, and also caused the death of a child [53]. A possible explanation for this outbreak is the reactivation of ancient anthrax carcass burial sites in permafrost soil that had thawed as a result of unusually warm temperatures during the summer of 2016 [49]. Previously, there has been an even larger outbreak in the Yamal Peninsula reindeer population with 6700 dead animals reported in 1941. Possibly, much older viable spores could be retrieved from permafrost samples of high northern latitudes such as Siberia [5]. Various other examples for the extended soil-borne endurance of *B. anthracis* spores are compiled in [49].

Even countries where anthrax has become a very rare disease still bear a residual risk that excavation work at anthrax soil foci or potentially contaminated industrial sites will release *B. anthracis* spores concomitantly with soil and dust, thereby triggering anthrax infections. Exposure to spore-contaminated soil or water (i.e., waste disposal and wastewater) from areas of former tanneries, leather factories, and knackering yards has long been assumed to constitute a potential risk of infection. Therefore, special biosafety rules apply in German civil engineering to protect against possible infections caused by anthrax spores at such sites. According to these rules, property developers are usually required by the responsible health authorities to have the soil analyzed for anthrax spores before excavation work begins at potentially contaminated sites. Today, molecular methods (PCR) facilitate the detection of minute amounts of spores in soil. The question remains though whether small numbers of spores (near the detection limit) do really translate into a real risk of infection from such sites [49]. Through obtaining positive PCR signals from soil core samples examined in this study (Figure 1), we have repeatedly failed to isolate *B. anthracis* bacteria by classical plating. Typically, this approach is sufficient for anthrax pathogen isolation from soil or animal-product-processing facilities [10] if there is circumstantial evidence for anthrax [25,27]. In our study, pure cultures of *B. anthracis* could only be grown from the PCR-positive samples taken from soil cores when resorting to magnetic separation because of interference from closely related bacteria.

Of note, the two strains A042 and A080 that have been isolated from diseased cattle in Germany were found to be phylogenetic descendants of new isolates (Lux2x, Lux7, and Lux8) from the sampled tannery site. It is unlikely that there is a direct outbreak link between these strains. Instead, it is probable that both share a common ancestry in the core distribution regions of the Sterne clade, which may be located in northern China, possibly Inner Mongolia. In this model, trade between China, the German concession harbors in China, and the German Reich and its neighbors (including Luxembourg) has spread spores to Central Europe. Unfortunately, this model is not supported by the incomplete documentation of the now abandoned tannery. These records state that the hides processed there had been imported from South, Central, and North America; from Africa (countries were not specified); and from Australia and New Zealand, as well as from neighboring countries (Belgium and France, including Corsica). Besides missing documentation on imports from China, one may also hypothesize that spores were introduced to this site by processing contaminated animal products from neighboring countries in which the Asian A.Br.075 *B. anthracis* spores have caused local anthrax outbreaks. In any case, if the (incomplete) tannery documentation is correct on the origin of the processed animal products, we should be able to isolate other genotypes (from A- and B-branch) when resampling this site. Notwithstanding the spores’ origin, it is noteworthy that all Lux isolates still carry a full set of virulence plasmids, even despite their long dormancy in soil. This was also the case for those strains collected from a wool processing facility in Belgium [10].

*B. anthracis* isolated from soil has frequently lost one or both virulence plasmids [32,54,55]. The reasons for this loss are unclear but could occur within the infected host body or after the spores’ release into soil. In this respect, the new tannery soil isolates described here are not untypical as none of the genotyped Lux isolates have lost any of their plasmids. While we and others have hypothesized on a soil-borne life-cycle of *B. anthracis* in the past [32,56,57], this finding rather supports a different, more inert view of soil-borne *B. anthracis* spores [58].

## 5. Conclusions

The prominent major canSNP clade A.Br.075(Sterne) of *B. anthracis* is replete with derivatives of the Sterne live vaccine strain. Nevertheless, this lineage also comprises numerous fully virulent and diverse members. We have now added a variety of archival strains that fill in phylogenetical gaps within this clade and provided unexpected new isolates from a long-time abandoned tannery. While the likely natural habitat of the A.Br.075(Sterne) clade is Central Asia, the isolation of members of this clade in other parts of the world likely stems both from historical anthropogenic dispersal of this branch and the broad use of the Sterne live vaccine in husbandry.

## Figures and Tables

**Table 1:** Initial DMAA PCR-based canSNP-typing of *B. anthracis* soil sample isolates.

DMAA-Test ^1^	Strain	Δ(Ct_DER_–Ct_ANC_) ^2^	SNP-Allele	canSNP Group
canSNP A.Br.001 (A.Br.Ames)	Lux1-5, Lux7, Lux10, Lux11, Lux1x-5x, Lux7x, Lux8x	19.2 ± 0.5	ANC	Non-A.Br.001
	A.Br.001 strains	−13.4 ± 4.0	DER	A.Br.001
	A.Br.002 strains	17.2 ± 2.5	ANC	Non-A.Br.001
				
canSNP A.Br.002 (A.Br.Sterne)	Lux1-5, Lux7, Lux10, Lux11, Lux1x-5x, Lux7x, Lux8x	−10.3 ± 0.7	DER	A.Br.002
	A.Br.002 strains	−10.2 ± 0.8	DER	A.Br.002
	Other A.Br. strains *	9.9 ± 0.4	ANC	Non-A.Br.002

^1^ canSNPs A.Br.001 and A.Br.002 [21]; ^2^ combined average values (and standard deviations) of several strains from the indicated canSNP groups of strains. * excluding A.Br.001 strains.

**Table 2:** New DMAA PCR assays for SNP-typing of *B. anthracis* A.Br.075 sub-clades.

DMAA-Test *	Strain Name	Δ(Ct_DER_ –Ct_ANC_) **	SNP Allele	(can)SNP Group
Sub-clade ‘Ortho-Sterne’ ^1^ (position identical to A.Br.076 ***)	Lux2-5, Lux7, Lux10, Lux11, Lux1x-5x, Lux7x, Lux8x, A034, A037, A040, A066	−13.2 ± 2.4	DER	‘Ortho-Sterne’
	Lux1, Ames, Sterne(A118), A014, A031, A103, A151, A153, A154, A210	14.7 ± 2.2	ANC	Non-‘Ortho-Sterne’
				
Sub-clade ‘Para-Sterne’ ^2^ (position identical to A.Br.078 ***)	A095, A102, A107, A81, CVI 131959, Sirajganj-2, A103	−11.3 ± 2.1	DER	‘Para-Sterne’
	Lux1-5, Lux7, Lux10, Lux11, Lux1x-5x, Lux7x, Lux8x, Sterne(A118), A100, A106, A014, A031, A151, A153, A154, A210	11.3 ± 1.9	ANC	Non-‘Para-Sterne’
				
Sub-clade ‘Eu-Sterne’ ^3^	Lux1, Sterne(A118), A31, A100, A106, A123, A065, A178	−6.5 ± 1.9	DER	‘Eu- Sterne’
	Lux2-5, Lux7, Lux10, Lux11, Lux1x-5x, Lux7x, Lux8x, A014, A015, A103, A106, A151, A153, A154, A210, 2862, Sirajganj-2	12.5 ± 2.2	ANC	Non-‘Eu-Sterne’
				
Sub-clade A.Br.079 ***^4^	Lux1, A015, A100	−13.7 ± 3.2	DER	A.Br.079
	Lux2x, A106, Sirajganj-2	11.6 ± 2.9	ANC	Non-A.Br.079
				
Sub-clade A.Br.080 ***^5^	Lux1, A065	−11.3 ± 0.6	DER	A.Br.080
	Lux2x, Sterne(A118)	10.8 ± 0.9	ANC	Non-A.Br.080
				
Sub-clade A02/B1+NL ****^6^	Sirajganj-2, CVI 13185	−8.8 ± 2.1	DER	A02/B1+NL
	Sterne(A118), 2865, Tangail-3	9.8 ± 0.7	ANC	Non- A02/B1+NL
				
Sub-clade SNP 4212929 ^7^	A100, A118, AF7, AF18	−11.6 ± 0.3	DER	4212929
	A015, A031	13.6 ± 5.0	ANC	Non- 4212929
				
Sub-clade SNP 5204006 ^8^	A015, A031	−8.5 ± 0.8	DER	5204006
	A100, AF7	8.6 ± 0.3	ANC	Non- 5204006

* SNPs at positions 1,198,831 ^1^, 2,658,086 ^2^, 5,140,491 ^3^, 571,199 ^4^, 2,271,412 ^5^, 3,766,679 ^6^, 4,212,929 ^7^, and 5,204,006 ^8^, in the reference chromosome NC_007530. ** Combined average values (and standard deviations) of several strains from the indicated canSNP groups of strains. *** [21]; **** [25].

## Data Availability

The genome sequence data presented in this study are available from the NCBI database under the BioProject ID: PRJNA309927. These and the accession numbers of publicly available genome sequences analyzed are listed in the Supplementary Materials (Supplementary Table S1) of this study.

## Supplementary Materials

The following supporting information can be downloaded at https://www.mdpi.com/article/10.3390/pathogens14010083/s1. Table S1: *Bacillus anthracis* strains analyzed in this study. Table S2: Primers used for DMAA analysis. Table S3: SNP matrix of analyzed *Bacillus anthracis* chromosomes. references [13,17,21,25,30] are cited in Supplementary Materials.
